# Excessive White Matter Hyperintensity Increases Susceptibility to Poor Functional Outcomes After Acute Ischemic Stroke

**DOI:** 10.3389/fneur.2021.700616

**Published:** 2021-09-10

**Authors:** Sungmin Hong, Anne-Katrin Giese, Markus D. Schirmer, Anna K. Bonkhoff, Martin Bretzner, Pamela Rist, Adrian V. Dalca, Robert W. Regenhardt, Mark R. Etherton, Kathleen L. Donahue, Marco Nardin, Steven J. T. Mocking, Elissa C. McIntosh, John Attia, Oscar R. Benavente, John W. Cole, Amanda Donatti, Christoph J. Griessenauer, Laura Heitsch, Lukas Holmegaard, Katarina Jood, Jordi Jimenez-Conde, Jaume Roquer, Steven J. Kittner, Robin Lemmens, Christopher R. Levi, Caitrin W. McDonough, James F. Meschia, Chia-Ling Phuah, Arndt Rolfs, Stefan Ropele, Jonathan Rosand, Tatjana Rundek, Ralph L. Sacco, Reinhold Schmidt, Christian Enzinger, Pankaj Sharma, Agnieszka Slowik, Alessandro Sousa, Tara M. Stanne, Daniel Strbian, Turgut Tatlisumak, Vincent Thijs, Achala Vagal, Johan Wasselius, Daniel Woo, Ramin Zand, Patrick F. McArdle, Bradford B. Worrall, Ona Wu, Christina Jern, Arne G. Lindgren, Jane Maguire, Liisa Tomppo, Polina Golland, Natalia S. Rost, The MRI-GENIE and GISCOME Investigators and the International Stroke Genetics Consortium, Sungmin Hong

**Affiliations:** ^1^J. Philip Kistler Stroke Research Center, Massachusetts General Hospital and Harvard Medical School, Boston, MA, United States; ^2^Department of Neurology, University Medical Center Hamburg-Eppendorf, Hamburg, Germany; ^3^Clinic for Neuroradiology, University Hospital Bonn, Bonn, Germany; ^4^Univ. Lille, Inserm, CHU Lille, U1172 - LilNCog (JPARC) - Lille Neurosciences & Cognition, Lille, France; ^5^Division of Preventive Medicine, Department of Medicine, Brigham and Women's Hospital and Harvard Medical School, Boston, MA, United States; ^6^Computer Science and Artificial Intelligence Lab, Massachusetts Institute of Technology, Boston, MA, United States; ^7^Athinoula A. Martinos Center for Biomedical Imaging, Department of Radiology, Massachusetts General Hospital, Charlestown, MA, United States; ^8^Hunter Medical Research Institute, Newcastle, NSW, Australia; ^9^School of Medicine and Public Health, University of Newcastle, Newcastle, NSW, Australia; ^10^Division of Neurology, Department of Medicine, University of British Columbia, Vancouver, BC, Canada; ^11^Department of Neurology, University of Maryland School of Medicine and Veterans Affairs Maryland Health Care System, Baltimore, MD, United States; ^12^School of Medical Sciences, University of Campinas (UNICAMP) and the Brazilian Institute of Neuroscience and Neurotechnology (BRAINN), Campinas, Brazil; ^13^Department of Neurosurgery, Geisinger, Danville, PA, United States; ^14^Research Institute of Neurointervention, Paracelsus Medical University, Salzburg, Austria; ^15^Division of Emergency Medicine, Washington University School of Medicine, St. Louis, MO, United States; ^16^Department of Neurology, Washington University School of Medicine & Barnes-Jewish Hospital, St. Louis, MO, United States; ^17^Department of Clinical Neuroscience, Institute of Neuroscience and Physiology, The Sahlgrenska Academy, University of Gothenburg and Department of Neurology, The Sahlgrenska University Hospital, Gothenburg, Sweden; ^18^Department of Neurology, Neurovascular Research Group (NEUVAS), IMIM-Hospital del Mar (Institut Hospital del Mar d'Investigacions M‘ediques), Universitat Autonoma de Barcelona, Barcelona, Spain; ^19^KU Leuven - University of Leuven, Department of Neurosciences, Experimental Neurology and Leuven Research Institute for Neuroscience and Disease (LIND), Leuven, Belgium; ^20^VIB, Vesalius Research Center, Laboratory of Neurobiology, University Hospitals Leuven, Department of Neurology, Leuven, Belgium; ^21^Department of Neurology, John Hunter Hospital, Newcastle, NSW, Australia; ^22^Department of Pharmacotherapy and Translational Research and Center for Pharmacogenomics, University of Florida, Gainesville, FL, United States; ^23^Department of Neurology, Mayo Clinic, Jacksonville, FL, United States; ^24^Centogene AG, Rostock, Germany; ^25^Department of Neurology, Clinical Division of Neurogeriatrics, Medical University Graz, Graz, Austria; ^26^Center for Genomic Medicine, Massachusetts General Hospital, Boston, MA, United States; ^27^Department of Neurology and Evelyn F. McKnight Brain Institute, Miller School of Medicine, University of Miami, Miami, FL, United States; ^28^Institute of Cardiovascular Research, Royal Holloway University of London (ICR2UL), Egham, United Kingdom; ^29^St. Peter's and Ashford Hospitals, Egham, United Kingdom; ^30^Department of Neurology, Jagiellonian University Medical College, Krakow, Poland; ^31^Department of Neurology, Helsinki University Hospital and Clinical Neurosciences, University of Helsinki, Helsinki, Finland; ^32^Department of Clinical Neuroscience, Institute of Neuroscience and Physiology, Sahlgrenska Academy at University of Gothenburg, Gothenburg, Sweden; ^33^Department of Neurology, Sahlgrenska University Hospital, Gothenburg, Sweden; ^34^Stroke Division, Florey Institute of Neuroscience and Mental Health, Heidelberg, VIC, Australia; ^35^Department of Neurology, Austin Health, Heidelberg, VIC, Australia; ^36^Department of Radiology, University of Cincinnati College of Medicine, Cincinnati, OH, United States; ^37^Department of Clinical Sciences Lund, Radiology, Lund University, Lund, Sweden; ^38^Department of Radiology, Neuroradiology, Skåne University Hospital, Malmo, Sweden; ^39^Department of Neurology and Rehabilitation Medicine, University of Cincinnati College of Medicine, Cincinnati, OH, United States; ^40^Department of Neurology, Geisinger, Danville, PA, United States; ^41^Division of Endocrinology, Diabetes and Nutrition, Department of Medicine, University of Maryland School of Medicine, Baltimore, MD, United States; ^42^Departments of Neurology and Public Health Sciences, University of Virginia, Charlottesville, VA, United States; ^43^Department of Laboratory Medicine, Institute of Biomedicine, The Sahlgrenska Academy, University of Gothenburg, Gothenburg, Sweden; ^44^Department of Neurology and Rehabilitation Medicine, Skåne University Hospital, Lund, Sweden; ^45^Department of Clinical Sciences Lund, Neurology, Lund University, Lund, Sweden; ^46^School of Nursing and Midwifery, University of Technology Sydney, Sydney, NSW, Australia

**Keywords:** white matter hyper intensity, stroke, brain health, brain vulnerability, post-stroke outcomes, functional independence, functional outcome after acute stroke, acute ischemic stroke

## Abstract

**Objective:** To personalize the prognostication of post-stroke outcome using MRI-detected cerebrovascular pathology, we sought to investigate the association between the excessive white matter hyperintensity (WMH) burden unaccounted for by the traditional stroke risk profile of individual patients and their long-term functional outcomes after a stroke.

**Methods:** We included 890 patients who survived after an acute ischemic stroke from the MRI-Genetics Interface Exploration (MRI-GENIE) study, for whom data on vascular risk factors (VRFs), including age, sex, atrial fibrillation, diabetes mellitus, hypertension, coronary artery disease, smoking, prior stroke history, as well as acute stroke severity, 3- to−6-month modified Rankin Scale score (mRS), WMH, and brain volumes, were available. We defined the unaccounted WMH (uWMH) burden *via* modeling of expected WMH burden based on the VRF profile of each individual patient. The association of uWMH and mRS score was analyzed by linear regression analysis. The odds ratios of patients who achieved full functional independence (mRS < 2) in between trichotomized uWMH burden groups were calculated by pair-wise comparisons.

**Results:** The expected WMH volume was estimated with respect to known VRFs. The uWMH burden was associated with a long-term functional outcome (β = 0.104, *p* < 0.01). Excessive uWMH burden significantly reduced the odds of achieving full functional independence after a stroke compared to the low and average uWMH burden [OR = 0.4, 95% CI: (0.25, 0.63), *p* < 0.01 and OR = 0.61, 95% CI: (0.42, 0.87), *p* < 0.01, respectively].

**Conclusion:** The excessive amount of uWMH burden unaccounted for by the traditional VRF profile was associated with worse post-stroke functional outcomes. Further studies are needed to evaluate a lifetime brain injury reflected in WMH unrelated to the VRF profile of a patient as an important factor for stroke recovery and a plausible indicator of brain health.

## Introduction

The ability of the brain to recover after an acute ischemic stroke (AIS) is linked to the pre-stroke burden of white matter hyperintensity (WMH), a neuroradiological biomarker of brain health (1–6). However, there is a significant variability in functional outcomes after AIS in association with WMH burden. It has not been fully characterized taking into consideration multiple clinical characteristics, such as age, sex, and other vascular risk factors (VRFs), that comprise a “traditional” stroke risk profile. Because those characteristics might be associated with stroke outcomes and also with accumulating WMH burden, quantifying the WMH burden effect on functional outcomes independent of clinical characteristics is challenging (2, 7–14). In addition to and compounded by age, a VRF profile is linked to overall brain health as reflected in the WMH burden—for example, an older patient with a history of hypertension is likely to have a larger WMH volume and less likely to achieve functional independence after AIS (2, 15). Prior studies using standard multiple regression models had a limited success in characterizing and quantifying an independent association between conventionally measured WMH volume and post-stroke outcome because the effects of confounding VRFs to the association of WMH and post-stroke outcomes were not explicitly accounted for (1, 2, 8, 9, 11, 12, 14, 15).

In this study, we hypothesized that the excessive portion of WMH burden unaccounted for by the clinical characteristics of an individual patient was an important indicator of brain health: i.e., the unaccounted WMH (uWMH) burden would show the clear association of the lifelong chronic brain damage independent of the VRF profile of a patient and the post-stroke functional outcomes, especially in achieving full functional independence after stroke. To test this hypothesis, we first defined the uWMH burden given the VRF profiles of patients to extrinsically quantify the portion of WMH burden that was independent of the VRF profiles. We then analyzed the uWMH association with post-stroke functional outcomes leveraging a large international multi-site cohort study for AIS, the MRI-Genetics Interface Exploration (MRI-GENIE), for the analyses and validation (16, 17).

## Methods

### Standard Protocol Approvals, Registrations, and Patient Consents

The image and clinical data were acquired from the MRI-GENIE study (16). The MRI-GENIE study is a large international cohort study of 19 sites, contributing 6,600 AIS patients with phenotypic, radiographic, and genotypic data. Patients were recruited in a hospital-based setting through Stroke Genetic Network (SiGN). The informed and written consent forms, including sharing of de-identified demographic, clinical phenotypic, and imaging data, were obtained from all patients or their legally authorized representative (18, 19). Each site received the approval of their internal review board.

### Data, Clinical Characteristics, and WMH Quantification

Age, sex, atrial fibrillation (AF), diabetes mellitus (DM), hypertension (HTN), coronary artery disease (CAD), smoking (SMK), and prior stroke history (PS) were included as the clinical characteristics of patients. Each vascular risk factor was recorded as a binary variable at hospital admission. The acute stroke severity of a patient was measured according to NIH Stroke Scale (NIHSS) at hospital admission.

T2 FLAIR MR images were obtained within the first 24 h from symptom onset. A more detailed description of the study can be found in previous publications (16, 20). White matter hyperintensity volume (WMHv) was quantified by a previously described automatic WMH segmentation framework (20). The framework consists of an automatic whole-brain extraction, intensity normalization, and WMH segmentation that leverages a deep neural network specifically for clinical grade T2-FLAIR images. Total brain volume was quantified automatically by a similar previously described framework (21). Both WMH and total brain segmentation results were evaluated, and the quality was controlled by experts and experienced raters. WMHv was adjusted by the total brain volume and log-transformed to correct its skewed distribution. The brain-volume-adjusted and log-transformed WMHv will be denoted as WMHv for simplicity in the rest of this paper.

**Figure 1:**
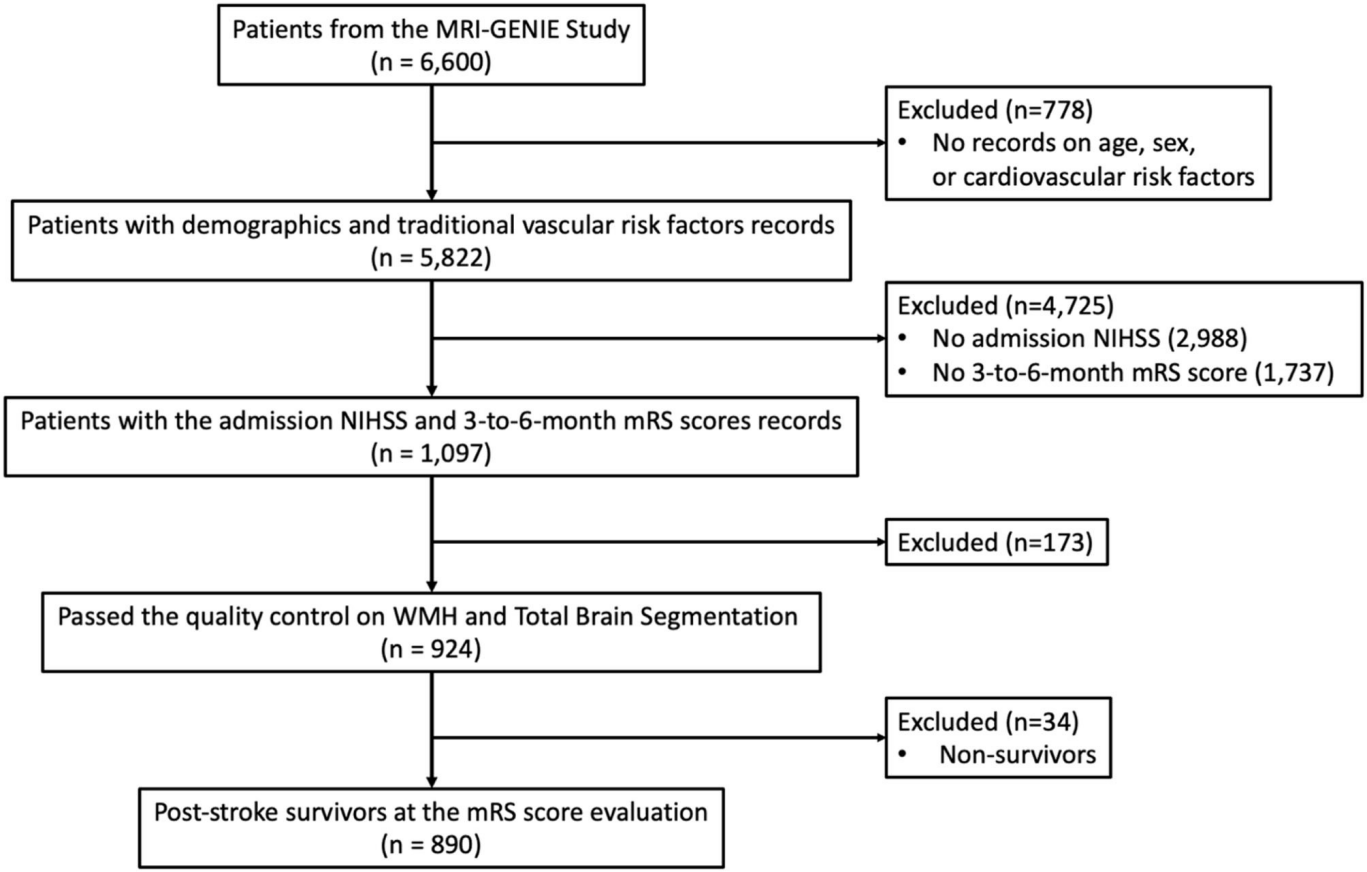
Flow chart on patient selection and its criteria.

Out of 6,600 patients, the complete clinical characteristics of 5,822 patients were reported. Acute stroke severity and long-term functional outcome data were available for 1,097 patients out of those 6,015 patients and measured by the acute NIHSS and the 3- to 6-month modified Rankin Scale (mRS) scores, respectively. After the quality control of WMH and total brain segmentation, 924 AIS patients remained. Thirty-four patients who did not survive after stroke at the time of the mRS score evaluation were excluded from the analysis, which resulted in a total of 890 patients. The patient selection and the criteria are summarized in Figure 1.

### Unaccounted WMH Burden

To measure uWMH independent of the VRF profile of a patient, we first modeled the association of WMH burden and the profile of the patient by multiple log-linear regression. Age, sex, AF, DM, HTN, CAD, SMK, and PS were considered in the analysis as explanatory variables, and WMHv was the target variable. NIHSS was included in the analysis as well to account for the effect of acute stroke severity on functional stroke outcomes for later analyses. The normality of WMHv data of the AIS population with respect to the multiple log-linear regression model was tested by the Shapiro–Wilks test.

The expected burden of WMH with respect to the VRF profile of a patient was calculated as the trend of the multiple log-linear regression model. The uWMH burden measured the amount of WMH burden that was not accounted for by the clinical profile of a patient, i.e., the residual WMH burden of the multiple log-linear regression model—for example, a positive uWMH value indicated that a patient had excessive WMHv than expected from her or his clinical profile, and negative means lower WMHv than expected. The normality of uWMH burden was tested by the Shapiro–Wilks test.

We analyzed the continuous association of uWMH burden and post-stroke functional outcome measured by the 3- to 6-month mRS score using the univariate linear regression model for the continuous association between uWMH burden and post-stroke functional outcomes.

We investigated how uWMH affected the possibility of functional independence after stroke by trichotomized analysis. We trichotomized patients into low, expected, and excessive eMWH burden groups based on uWMH burden. We defined the average group as patients within ±1 standard deviation from the mean uWMH burden, which resulted in 68% of total patients. The low and excessive uWMH burden groups were defined as lower and higher 16% of patients of the uWMH burden distribution, respectively.

The dichotomized 3- to 6-month mRS score (mRS excellent: mRS score 0–1, mRS moderate–poor: mRS score 2–6) was used to measure functional independence after AIS. We dichotomized the mRS scores to mRS excellent and mRS moderate–poor to investigate the association of uWMH burden and full functional independence after stroke. We calculated the pair-wise odds ratios of patients who achieved full functional independence for patients between all burden groups (e.g., low vs. expected, low vs. excessive, and expected vs. excessive). The 95% confidence interval and the statistical significance of the odds ratio were calculated by the standard *z*-test to investigate if there was a significant difference between the groups in achieving functional independence after stroke.

We performed the subgroup analysis regarding acute stroke severity by dividing the patients into mild and severe acute stroke severity groups. We divided the patients with respect to their acute NIHSS scores, with a threshold defined as the third quartile (Q3) of the NIHSS scores of the population. We performed the same trichotomized analysis of uWMH burden and investigated the odds ratios of patient who achieved full functional independence, defined as “excellent mRS” (with residual non-disabling symptoms, mRS 1, or symptom-free, mRS 0) between the uWMH burden groups of each acute severity subgroup.

All statistical analyses were performed with Python SciPy 1.5.4 (22), Sci-kit Learn 0.23 (23), and StatsModel 0.12 packages (24).

## Results

### Population Analysis

The population characteristics of the included patients are summarized in the first column of Table 1. The multiple log-linear regression model of WMHv with respect to all included clinical characteristics (age, sex, AF, HTN, DM, CAD, PS, and NIHSS) represented an expected WMHv, **Ŷ**_**WMHv**_, of a patient with respect to the VRF profile of the patient. The regression coefficients of the multiple log-linear regression model are summarized in Table 2. The multiple log-linear model explained 43.3% of the total variation of WMHv (*R*^2^ = 0.433). The uWMH burden was not correlated with any included clinical characteristics: i.e., the correlation coefficients of the uWMH burden to all included clinical characteristics were 0. The uWMH burden passed the normality test by the Shapiro–Wilks test (*p* < 0.001). The uWMH burden showed a significant association with continuous functional outcomes (3-month to 6-month mRS score: 0–5) in the univariate linear regression model β = 0.104, *p* < 0.01.

**Table 1:** Population clinical characteristics of total acute ischemic stroke patients from the MRI-GENIE study and those included the analysis with available admission NIHSS, 3- to 6-month mRS scores and WMHv, and the low, expected, and excessive uWMH burden groups.

	**Total (*n* = 5,822)**	**Total included in the analysis (*n* = 890)**	**Low (*n* = 140)**	**Expected (*n* = 599)**	**Excessive (*n* = 151)**
Age, average (SD)	61.3 (15.2)	63.7 (15.0)	61.6 (14.1)	64.1 (15.5)	64.2 (13.9)
Sex, *n* (%)	2,416 (41.5%)	379 (42.6%)	59 (42.1%)	251 (41.9%)	69 (45.7%)
HTN, *n* (%)	3,849 (66.1%)	541 (60.8%)	88 (62.9%)	355 (59.3%)	98 (64.9%)
DM, *n* (%)	1,480 (25.4%)	180 (20.2%)	24 (17.1%)	131 (21.9%)	25 (16.6%)
AF, *n* (%)	741 (12.7%)	140 (15.7%)	19 (13.6%)	98 (16.4%)	23 (15.2%)
CAD, *n* (%)	1,021 (17.5%)	140 (15.7%)	13 (9.3%)	105 (17.5%)	22 (14.6%)
SMK, *n* (%)	3,342 (57.4%)	475 (53.4%)	70 (50.0%)	331 (55.3%)	74 (49.0%)
PS, *n* (%)	749 (12.9%)	85 (9.6%)	13 (9.3%)	59 (9.8%)	13 (8.6%)
NIHSS, median (IQR)	N/A	3 (5)	3 (4)	3 (6)	3 (5)
uWMH, avg (std)	N/A	0 (1.1)	−1.8 (0.5)	0.0 (0.6)	1.6 (0.4)

**Table 2:** The regression coefficients of individual univariate and multiple log-linear regression analyses that modeled the associations of age, sex, VRFs, and NIHSS with WMHv.

***Y* = WMHv**	**Age**	**Sex**	**HTN**	**AF**	**CAD**	**DM**	**SMK**	**PS**	**NIHSS**	**β_0_**
Regression coefficients	0.06	−0.19	0.33	−0.01	−0.1	0.27	0.30	0.33	0.01	−9.82
*p*	<0.01	0.01	<0.01	0.96	0.4	<0.01	<0.01	0.01	0.08	<0.01

The second to the fourth columns of Table 1 summarize the population characteristics of the low, expected, and excessive uWMH burden groups. The averages of the uWMH burden of low, expected, and excessive eWMH burden groups were −1.8, 0.0, and 1.6, respectively.

**Figure 2:**
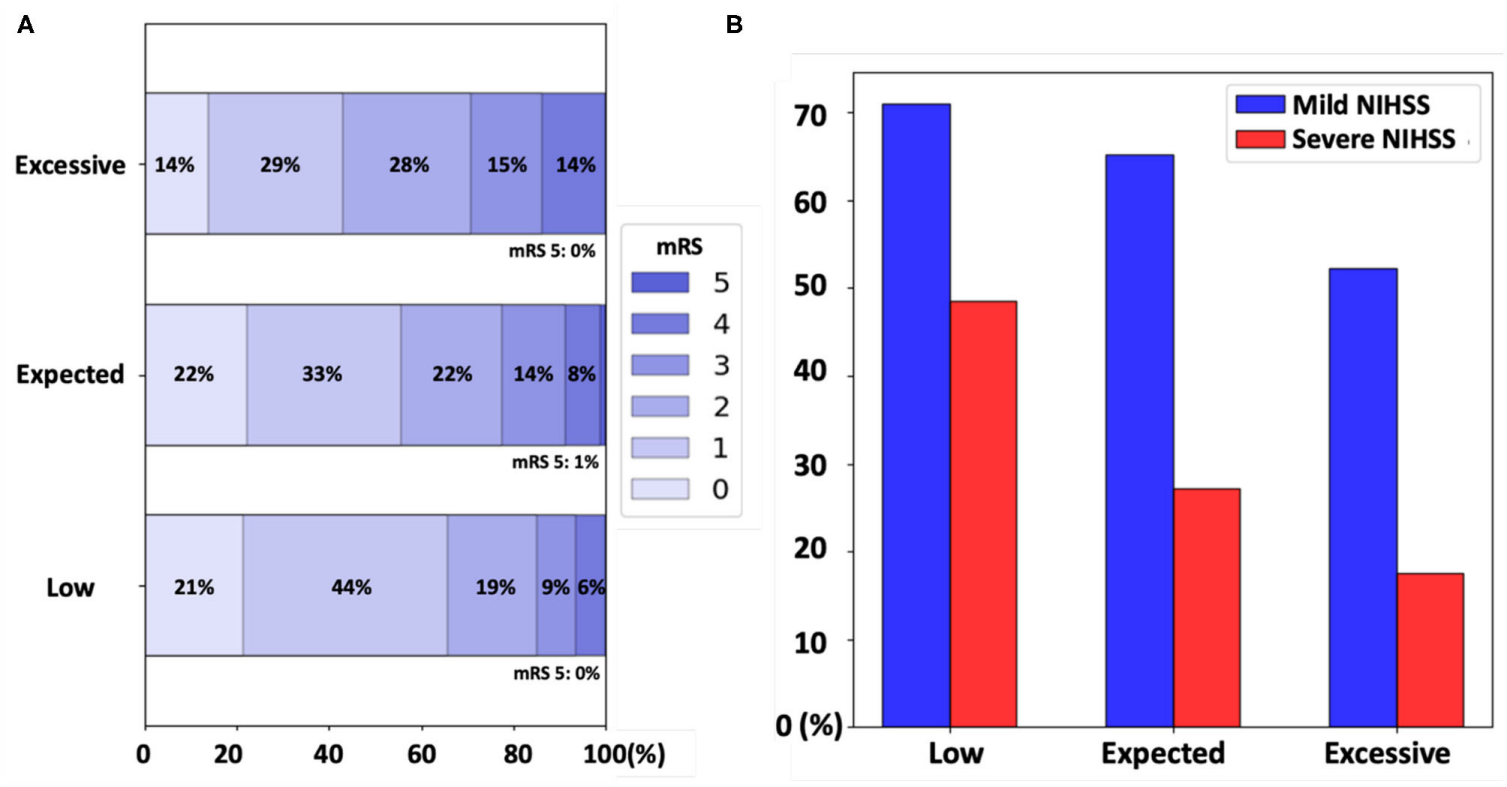
**(A)** The distribution of the modified Rankin Scale (mRS) scores of patients in the low, expected, and excessive unaccounted white matter hyperintensity (uWMH) burden groups. The proportions of mRS scores were shifted toward worse mRS scores for the higher uWMH burden groups. **(B)** The acute stroke severity subgroup analysis. The proportion of patients who achieved the full functional independence (mRS excellent: mRS 0 and 1) in each uWMH burden group was plotted in blue and in red for the mild [NIH Stroke Scale (NIHSS) <7] and severe (NIHSS ≥7) acute stroke severity groups, respectively.

The ratios of patients who achieved full functional independence (mRS excellent: mRS score 0–1) in the low, expected, and excessive uWMH burden groups were 65.7, 55.6, and 43%, respectively. The odds ratios of the mRS excellent patients between the uWMH burden groups are summarized in Table 3. Patients in the excessive uWMH burden groups were less likely to achieve full functional independence after AIS than the expected and excessive uWMH burden groups. The odds ratios of the excessive uWMH group with respect to the expected and low uWMH burden groups were 0.604 (*p* < 0.01) and 0.394 (*p* < 0.01), respectively. The odds ratio of the expected uWMH burden group with respect to the low group was 0.653 (*p* = 0.03). Figure 2A shows the distributions of mRS scores of the low, expected, and excessive uWMH burden groups. The distribution of mRS scores of the excessive uWMH burden groups was consistently shifted toward worse mRS scores compared to the lower uWMH groups.

**Table 3:** The odds ratios of patients who achieved full functional independence (mRS excellent: mRS scores 0–1) of the excessive uWMH burden groups to the low (excessive/low) and expected (excessive/expected) groups and of the expected group to the low (expected/low) group.

**Population**	**Excessive/low**	**Excessive/expected**	**Expected/low**
OR	0.39	0.6	0.65
*p*, 95% CI	<0.01 (0.25, 0.63)	<0.01 (0.42, 0.87)	0.03 (0.44, 0.96)

As an auxiliary analysis, we conducted the same analyses with all patients, including 34 post-stroke non-survivors (mRS = 6). The results did not change substantially by including non-survivors. The uWMH burden showed a significant association with functional outcomes (3-month to 6-month mRS score: 0–6) in the univariate regression model, β = 0.091, *p* = 0.036. The odds ratios between the low, expected, and excessive uWMH burden groups did not change substantially from the analysis with post-stroke survivors only. The detailed results are reported in Supplementary Tables 1–3 in the Supplementary Materials.

### Acute Stroke Severity Subgroup Analysis

The third quartile of the NIHSS scores of the population was seven. The median NIHSS scores of the mild (NIHSS <7) and severe (NIHSS ≥7) acute stroke severity subgroups were 2 and 11, respectively. The overall population characteristics of the acute stroke severity subgroups are summarized in Table 4.

**Table 4:** Population clinical characteristics of acute ischemic stroke patients in the mild acute stroke severity group (mild, *NIHSS* < 7) and severe stroke severity group (severe, *NIHSS* ≥ 7).

	**Mild (*n* = 666)**	**Severe (*n* = 224)**
Age, average (SD)	63.4 (15.2)	64.6 (14.3)
Sex, *n* (%)	273 (41.0%)	106 (47.3%)
HTN, *n* (%)	408 (61.3%)	133 (59.4%)
DM, *n* (%)	134 (20.1%)	46 (20.5%)
AF, *n* (%)	86 (12.9%)	54 (24.1%)
CAD, *n* (%)	95 (14.3%)	45 (20.1%)
SMK, *n* (%)	367 (55.1%)	108 (48.2%)
PS, *n* (%)	63 (9.5%)	22 (9.8%)
NIHSS, median (IQR)	2 (3)	11 (7)
uWMH, average (SD)	0.0 (1.1)	0.0 (1.1)

In the mild acute stroke severity subgroup, the ratios of mRS excellent patients in the low, expected, and excessive uWMH burden groups were 71, 65.2, and 52.3%, respectively. The odds ratios of the mRS excellent patients in the excessive uWMH burden group with respect to the low and expected uWMH burden groups were 0.446 (*p* < 0.01) and 0.585 (*p* = 0.01), respectively. The odds ratio of the expected uWMH burden group compared to the low uWMH burden group was 0.763 (*p* = 0.25).

In the severe acute stroke severity subgroup, the ratios of mRS excellent patients in the low, expected, and excessive uWMH burden groups were 48.5, 27.2, and 17.5%, respectively. The odds ratios of the mRS excellent patients in the excessive and expected uWMH burden groups with respect to the low uWMH burden group were 0.225 (*p* < 0.01) and 0.396 (*p* = 0.02). The odds ratio of the excessive uWMH burden group compared to the expected uWMH burden group was 0.569 (*p* = 0.21). The odds ratios of the subgroup analysis are summarized in Table 5 and visualized in Figure 2B.

**Table 5:** Acute stroke severity subgroup analysis.

**Acute stroke severity group**		**Excessive/** **low**	**Excessive/** **expected**	**Expected/** **low**
Mild (*n* = 666)	OR	0.45	0.58	0.76
	*p*, 95% CI	<0.01 (0.26, 0.78)	0.01 (0.38, 0.89)	0.25 (0.48, 1.21)
Severe (*n* = 224)	OR	0.23	0.57	0.40
	*p*, 95% CI	<0.01 (0.08, 0.65)	0.21 (0.23, 1.39)	0.02 (0.18, 0.86)

*The odds ratios of patients who achieved full functional independence (mRS excellent: mRS scores 0–1) of the excessive uWMH burden groups to the low (excessive/low) and expected (excessive/expected) groups and of the expected group to the low (expected/low) group in the mild acute stroke severity group (mild, NIHSS < 7) and the severe acute stroke severity group (severe, NIHSS ≥ 7)*.

We also conducted an auxiliary analysis with all patients, including the post-stroke non-survivors for the subgroup analysis. The odds ratios and *p*-values between the low, expected, and excessive uWMH burden groups were not changed substantially for both mild and severe acute stroke severity patients. The detailed results are summarized in Supplementary Tables 4, 5 in the Supplementary Materials.

## Discussion

In this study, we analyzed the association of uWMH, i.e., the amount of WMH burden that is not explained by the traditional VRFs of a patient, and post-stroke functional outcome. Our results showed that, in stroke survivors, uWMH was significantly associated with the 3–6 months of functional outcomes. Furthermore, our population analysis showed that patients with higher uWMH burden were less likely to achieve full functional independence (mRS < 2) than patients with lower uWMH burden, while their clinical characteristics were similar. Our study supports the previous suggestions that WMH severity implicates more than the effects of demographics and traditional VRFs and shows the clear association of the uWMH burden and complete post-stroke functional recovery, with the uWMH burden emerging as a new independent variable to utilize in future analyses of post-stroke functional outcomes (25).

The consistent results of the trichotomized analysis with respect to the uWMH burden on the entire population showed that excessive uWMH burden was linked to less probability of achieving full functional independence and the opposite for low uWMH. The mean uWMH burden of patients in the low uWMH burden group was negative; thus, patients in the low uWMH group had lower WMH severity than expected from their demographics and traditional VRF profiles. This might indicate that those patients who were more resilient to a lifelong, chronic damage to the brain (as measured by WMH severity) were more likely to achieve full functional independence after stroke than those with similar VRF profiles but unexpectedly excessive uWMH. The consistent finding that patients in the excessive uWMH burden group were less likely to achieve full functional independence compared to those in the other groups also supports this interpretation.

The subgroup analysis based on acute stroke severity showed that the uWMH burden was linked to full functional independence for patients with both mild and severe acute stroke severity. Because our patient population had milder acute stroke severity (*median NIHSS* = 3) than a typical clinical scenario of mixed mild and severe strokes, separate subgroup analyses, including mild acute (*median NIHSS* = 2) and severe (*median NIHSS* = 11) strokes, provided additional information on the uWMH association with full functional independence after stroke (mRS 0–1). The results were consistent with the overall population analysis; excessive uWMH burden in these subgroups resulted in a decreased probability of achieving full functional independence and the opposite for low uWMH.

In the severe stroke subgroup, patients with low uWMH showed significantly greater odds of achieving full functional independence after stroke than any others. In the mild stroke group, the odds of achieving full functional independence by patients with excessive uWMH was significantly lower compared to other groups. These findings might indicate that a patient with the brain resilient to chronic cerebrovascular injury is more robust to achieve full functional recovery even after severe stroke, while a patient with a vulnerable brain (as reflected by excessive uWMH) is more susceptible to post-stroke disability even after a mild stroke (as shown in Figure 2B). The additional finding to highlight with regard to these data is that the probability of achieving full functional independence post-stroke (mRS 0–1) in mild stroke patients with excessive uWMH and the probability of those with low uWMH burden and severe stroke were similar at 52.3 and 48.5%, respectively (Table 5 and Figure 2B). We interpret these data as potential evidence of the strong effect by uWMH on the functional outcome in patients with different levels of acute stroke severity. The excessive uWMH burden was more predictive on lower functional outcomes for those with mild stroke severity and, on the other hand, the low uWMH burden was more protective on achieving functional independence for those with severe stroke severity.

The uWMH burden was not correlated with any of the other included clinical characteristics. This was expected since uWMH represent residuals of the multiple log-linear regression model with respect to the clinical characteristics. The multiple log-linear regression modeling of WMHv and the other clinical characteristics and the definition of the uWMH burden as the residual of the model can be interpreted as the adjustment of the effects of the demographics and traditional VRF profile of a patient to the WMHv. The uWMH burden analysis enabled the explicit exploration of the association between WMH severity and post-stroke functional outcomes, free of the traditional VRF patient profile effect (26). We only included the univariate linear regression analysis for the uWMH association with the continuous post-stroke functional outcome because the uWMH burden was not correlated with the included clinical factors.

There are several limitations of our study that are worth considering for further investigations. The clinical characteristics that were considered in this analysis were not exhaustive of all known factors associated with WMH and stroke outcomes. WMH accumulation depends on many factors: normal aging (27), VRF aggregation, blood–brain barrier disruption (28), amyloid and tau accumulation (29), hyperlipidemia (8, 14), hyperhomocysteinemia (30), and genetics (31). Because the uWMH burden captured the effects of the demographics and traditional VRFs in a stroke cohort by modeling, it might have captured the effect of unmeasured risk factors, including the aforementioned VRFs, stroke etiologies, or genetics. The binary representations of the traditional VRFs may have undermined their effects on the WMH burden. The additional information on a time-based load of VRFs may improve the estimation of the expected WMH burden based on the clinical characteristics of the patients and the quantification of the unaccounted WMH burden. The education and personal lifestyle of a patient that may also be related to the WMH accumulation will improve the personalized analysis on the association of the WMH burden and functional stroke outcomes. The other possible risk factors will need to be investigated further by other studies that specifically focus on non-traditional risk factors for WMH and that may explain the remaining unexplained variance of functional outcomes in this study.

The post-stroke treatment information of patients was not included in the analysis because the information was not available in our study. The post-stroke treatment information will enable a stratified analysis on patients who received post-stroke treatments and those did not, with a careful assessment on the heterogeneity of post-stroke treatments and their complications. The cognitive decline of a patient will also provide an important insight on the interaction between the WMH burden and functional outcomes that can be investigated further.

Patients in the low uWMH burden group were slightly younger than the expected and excessive uWMH burden groups. Patients in the low uWMH burden groups were also less likely to have DM, AF, CAD, and smoking than the expected uWMH groups. Although the differences were not large, it might have affected the results. However, patients in the excessive uWMH burden group also had less proportions of having histories of DM, AF, CAD, and smoking, which might suggest that the differences were caused by the relatively small sample sizes of the low and excessive uWMH burden groups. It is worth to note that categorizing patients with respect to WMHv would have resulted in larger differences in clinical characteristic distribution across the groups and further complicated the interpretation on the association of WMH severity and post-stroke outcomes.

Being focused on explaining functional outcomes, our analysis does not expand to the predictive capacity of the uWMH burden for post-stroke functional outcomes. Our analysis was also not validated with an independent cohort because of the availability. The strong association of the uWMH burden with post-stroke functional outcomes suggests that the uWMH burden could be of benefit for future clinical prediction models of functional outcomes. We do not suggest the uWMH burden as the strongest predictor of post-stroke functional outcomes. As shown in the subgroup analysis regarding the acute stroke severity, the acute NIHSS was a stronger predictor of post-stroke functional outcomes. However, the subgroup analysis also showed that the uWMH burden could complement the prediction of functional outcomes with the admission NIHSS. This will be an interesting research direction to investigate further regarding the predictive capability of the uWMH burden, combined with other risk factors including the acute stroke severity, by using predictive modeling (e.g., logistic regression, decision forests, deep learning models, etc.). The predictive power of the uWMH burden and its association to functional outcomes will be benefited from cross-validation across multiple datasets and also from validation with a dataset from an independent cohort.

To enable the analysis of WMHv with the largest possible number of image data, we used the automatic segmentation framework to obtain WMHv. Although the WMH segmentation masks were validated quantitatively and qualitatively, we did not have access to old and new stroke lesion outlines and could hence not comprehensively account for either one (20). It is therefore possible that stroke infarcts with WMH similarity might have led to some inaccuracies in WMH delineation. This effect might be especially pronounced in patients with a high acute stroke severity and larger, or multiple, old and new stroke infarcts. However, in view of a median NIHSS of only three, the median size of stroke infarcts is expected to be small, as is their impact on WMHv estimation.

Lastly, WMHv and whole-brain volume were quantified by a scalar abstract measure—volume. Future studies should investigate how high-dimensional brain features, such as WMH, in relation to different white matter subregions and brain anatomical structures (e.g., white matter, striatal complex, and ventricle), are associated with functional post-stroke outcomes.

## Conclusion

The excessive burden of WMH that was not accounted for by the demographics and traditional VRFs of each individual patient is associated with post-stroke functional outcomes, especially with full functional independence after stroke. Our analysis suggests that a lifetime injury to the white matter that is not explained by traditional VRF profile is an important factor for stroke recovery and a plausible indicator of brain health. In the future, an individual risk profile of an AIS patient and its relationship to WMH should be considered in a personalized approach to post-stroke outcome prediction.

## Data Availability Statement

The original contributions presented in the study are included in the article/Supplementary Material, further inquiries can be directed to the corresponding author/s.

## MRI-GENIE, GISCOME and International Stroke Genetics Consortium

Sungmin Hong, Massachusetts General Hospital, Boston, MA, USA; Anne-Katrin Giese, Massachusetts General Hospital, Boston, MA, USA; Markus D. Schirmer, Massachusetts General Hospital, Boston, MA, USA; Anna K. Bonkhoff, Massachusetts General Hospital, Boston, MA, USA; Martin Bretzner, Massachusetts General Hospital, Boston, MA, USA; Pamela Rist, Brigham and Women's Hospital, Boston, MA, USA; Adrian V. Dalca, Massachusetts Institute of Technology, Cambridge, MA, USA; Robert W. Regenhardt, Massachusetts General Hospital, Boston, MA, USA; Mark R. Etherton, Massachusetts General Hospital, Boston, MA, USA; Kathleen L. Donahue, Massachusetts General Hospital, Boston, MA, USA; Marco Nardin, Massachusetts General Hospital, Boston, MA, USA; Steven J. T. Mocking, Massachusetts General Hospital, Boston, MA, USA; Elissa C. McIntosh, Massachusetts General Hospital, Boston, MA, USA; John Attia, University of New Castle, New South Wales, Australia; Oscar R. Benavente, University of British Columbia, Vancouver, British Columbia, Canada; John W. Cole, University of Maryland, Baltimore, MD, USA; Amanda Donatti, University of Campinas, Campinas, Brazil; Christoph J. Griessenauer, Geisinger, Danville, PA, USA; Laura Heitsch, Washington University, St. Louis, MO, USA; Lukas Holmegaard, University of Gothenburg, Gothenburg, Sweden; Katarina Jood, University of Gothenburg, Gothenburg, Sweden; Jordi-Jimenez-Conde, Universitat Autonoma de Barcelona, Barcelona, Spain; Jaume Roquer, Universitat Autonoma de Barcelona, Barcelona, Spain; Steven J. Kittner, University of Maryland, Baltimore, USA; Robin Lemmens, University of Leuven, Leuven, Belgium; Christopher R. Levi, Hunter Medical Research Institute, New Castle, South Wales, Australia; Caitrin W. McDonough, University of Florida, Gainesville, FL, USA; James F. Meschia, Mayo Clinic, Jacksonville, FL, USA; Chia-Ling Phuah, Washington University, St. Louis, MO, USA; Arndt Rolfs, Centogene AG, Rostock, Germany; Stefan Ropele, Medical University Graz, Graz, Austria; Jonathan Rosand, Massachusetts General Hospital, Boston, USA; Tatjana Rundek, University of Miami, Miami, USA; Ralph L. Sacco, University of Miami, Miami, USA; Reinhold Schmidt, Medical University Graz, Graz, Austria; Christian Enzinger, Medical University Graz, Graz, Austria; Pankaj Sharma, Royal Holloway University of London, Egham, UK; Agnieszka Slowik, Jagiellonian University, Krakow, Poland; Alessandro Sousa, University of Campinas, Campinas, SP, Brazil; Tara M. Stanne, University of Gothenburg, Gothenburg, Sweden; Daniel Strbian, Helsinki University Central Hospital, Helsinki, Finland; Turgut Tatlisumak, University of Gothenburg, Gothenburg, Sweden; Vincent Thijs, University of Melbourne, Heidelberg, Australia; Achala Vagal, University of Cincinnati, Cincinnati, OH, USA; Johan Wasselius, Skåne University, Lund, Sweden; Daniel Woo, University of Cincinnati, Cincinnati, OH, USA; Rasmin Zand, Geisinger, Danville, PA, USA; Patrick F. McArdle, University of Maryland, Baltimore, MD, USA; Bradford B. Worrall, University of Virginia, Charlottesville, VA, USA; Ona Wu, Massachusetts General Hospital, Boston, MA, USA; Christina Jern, University of Gothenburg, Gothenburg, Sweden; Arne Lindgren, Skåne University, Lund, Sweden; Jane Maguire, University of Technology Sydney, Sydney, Autralia; Liisa Tomppo, Helsinky University Central Hospital, Helsinki, Finland; Polina Golland, Massachusetts Institute of Technology, Cambridge, MA, USA; Natalia S. Rost, Massachusetts General Hospital, Boston, MA, USA.

## Author Contributions

SH, A-KG, MS, and NR contributed to conception and design of the study. SH performed statistical analysis and drafted the manuscript. SH, AB, MB, PR, and NR analyzed and reviewed the analysis and data. All authors did major roles in the acquisition of data, contributed to manuscript revision, read, and approved the submitted version.

## Funding

NIH-NINDS (MRI-GENIE: R01NS086905 - PI NR; K23NS064052, R01NS082285 - NR; SiGN: U01 NS069208 - JR, SK; R01NS059775, R01NS063925, R01NS082285, P50NS051343, R01NS086905, U01 NS069208 - OW); NIH NIBIB (NAC P41EB015902 PG, U01NS030678 Kissela, Kleindorfer; EB015325 OW); ISGS (R01NS423733 P.I. JM, Swedish Heart and Lung Foundation, Lund University, Region Skåne, the Freemasons Lodge of Instruction Eos Lund, Skåne University Hospital, the Foundation of Färs &Frosta (One of Sparbanken Skånes ownership Foundations), and the Swedish Stroke Association - AL, Swedish Research Council and the Swedish Heart and Lung Foundation, the Swedish State under the ALF agreement CJ, Spanish Ministry of Science and Innovation, Instituto de Salud Carlos III (Funding for Research in Health PI051737, PI10/02064, PI12/01238 and PI15/00451 JJ-C), Fondos FEDER/EDRF Red de Investigación Cardiovascular (RD12/0042/0020 JJ-C), Fundació la Marató TV3 (76/C/2011 - JJ-C) and Recercaixa13 (JJ086116 JJ-C), Wistron Corporation (PG); The European Unions Horizon 2020 research and innovation programme under the Marie Sklodowska-Curie grant agreement No 753896 - MS; RL is a senior clinical investigator of FWO Flanders; MB was supported by the ISITE-ULNE Fundation, the Société Française de Neuroradiologie, the Société Française de Radiologie, the Thérèse and René Planiol Fundation; ME was supported by the American Academy of Neurology and MGH Executive Council on Research; TT was supported by the Helsinki University Central Hospital, Sigrid Juselius Foundation, Sahlgrenska University Hospital, and University of Gothenburg. The funders were not involved in the study design, collection, analysis, interpretation of data, the writing of this article of the decision to submit it for publication.

## Conflict of Interest

AR was employed by Centogene AG, Germany. The remaining authors declare that the research was conducted in the absence of any commercial or financial relationships that could be construed as a potential conflict of interest.

## Publisher's Note

All claims expressed in this article are solely those of the authors and do not necessarily represent those of their affiliated organizations, or those of the publisher, the editors and the reviewers. Any product that may be evaluated in this article, or claim that may be made by its manufacturer, is not guaranteed or endorsed by the publisher.
